# Advancements in monkeypox vaccines development: a critical review of emerging technologies

**DOI:** 10.3389/fimmu.2024.1456060

**Published:** 2024-10-11

**Authors:** Igor Garcia-Atutxa, Paul Mondragon-Teran, Alejandro Huerta-Saquero, Francisca Villanueva-Flores

**Affiliations:** ^1^ Computer Science Department, Universidad Católica de Murcia (UCAM), Murcia, Spain; ^2^ Centro de Investigación en Ciencia Aplicada y Tecnología Avanzada (CICATA) Unidad Morelos del Instituto Politécnico Nacional (IPN), Xochitepec, Morelos, Mexico; ^3^ Departamento de Bionanotecnología, Centro de Nanociencias y Nanotecnología, Universidad Nacional Autónoma de México (UNAM), Ensenada, Mexico

**Keywords:** monkeypox, vaccine, orthopoxvirus, zoonosis, outbreak

## Abstract

Monkeypox (mpox) is a zoonotic illness caused by the monkeypox virus (MPXV), with higher health concerns among people who are pregnant, children, and persons who are immunocompromised, including people with untreated and advanced HIV disease. Significant progress has been made in developing vaccines against mpox, yet critical challenges and limitations persist in ensuring their effectiveness, safety, and accessibility. The pertinence of this review is highlighted by the World Health Organization’s declaration of a global health emergency on August 14, 2024, due to the recent mpox outbreak, underscoring the critical necessity for effective vaccine solutions in the face of a rapidly evolving virus. Here, we comprehensively analyze various vaccine platforms utilized in mpox prevention, including attenuated and non-replicating virus vaccines, viral vector-based vaccines, recombinant protein vaccines, and DNA and mRNA vaccines. We evaluate the advantages and limitations of each platform, highlighting the urgent need for ongoing research and innovation to enhance vaccine efficacy and safety. Recent advancements, such as incorporating immunostimulatory sequences, improved delivery systems, and developing polyvalent vaccines, are explored for their potential to offer broader protection against diverse orthopoxvirus strains. This work underscores the need to optimize currently available vaccines and investigate novel vaccination strategies to address future public health emergencies effectively. By focusing on these advanced methodologies, we aim to contribute to the development of robust and adaptable vaccine solutions for mpox and other related viral threats.

## 1 Introduction

**Figure 1:**
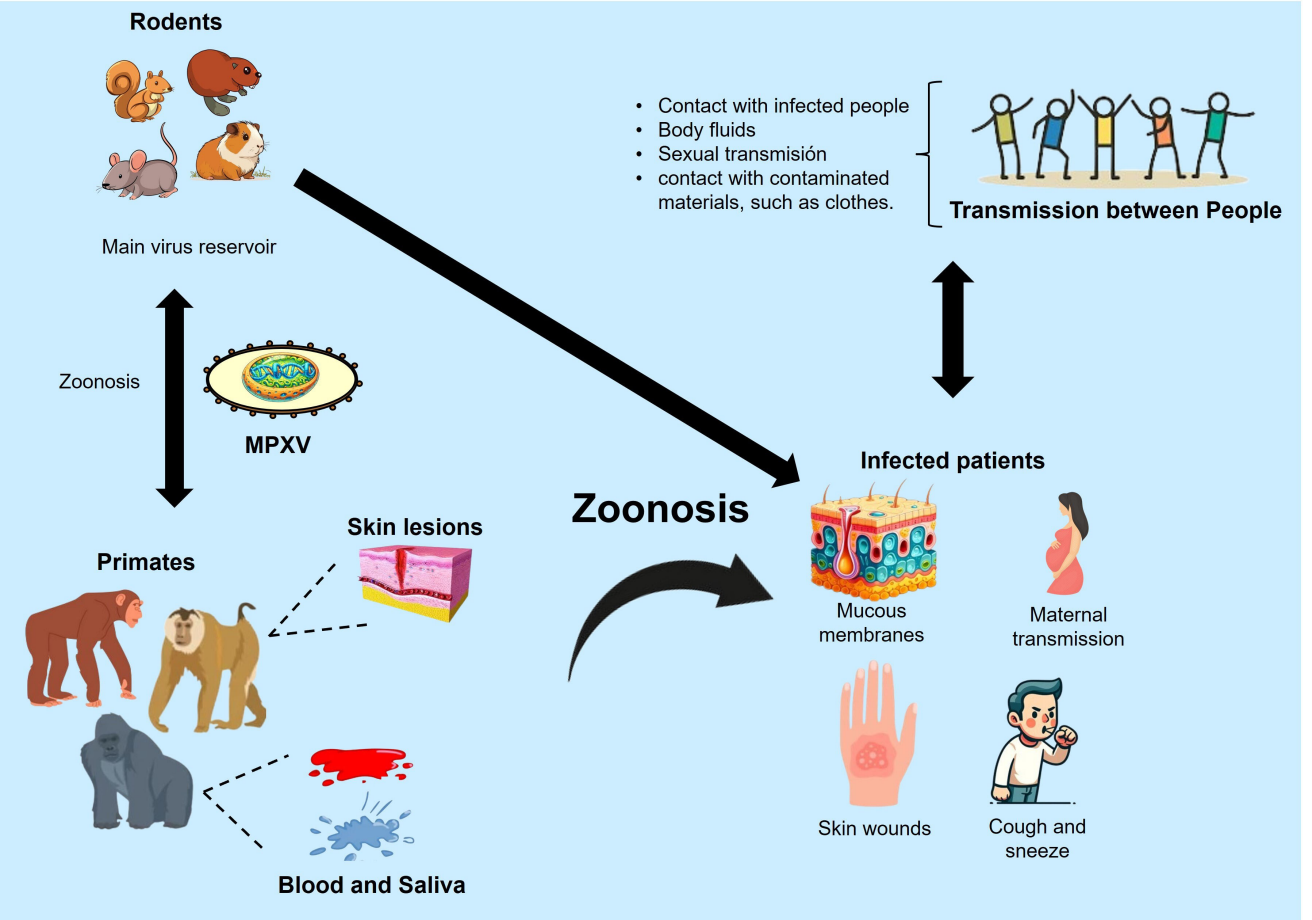
Zoonotic transmission mechanisms of MPXV. Modified from (106).

Monkeypox (mpox) is a zoonotic disease caused by the monkeypox virus (MPXV), which belongs to the Orthopoxvirus genus in the Poxviridae family (1). MPXV primary reservoirs are various species of African rodents, such as rope squirrels and rats of the genus Cricetomys. Through a zoonotic mechanism, the virus can be transmitted to primates via skin-to-skin contact, body fluids like blood or saliva, or sexual contact (**Figure 1**). The mpox disease manifests with symptoms similar to smallpox, though generally less severe, including fever, headache, muscle aches, and a distinctive rash that spreads across the body, reviewed in (2). The World Health Organization (WHO) has reclassified MPXV into Type I and Type II, formerly designated according to their endemic locations in Central and West Africa, respectively. This change in terminology aims to eliminate geographically discriminatory names (3). Type II MPXV is notably more virulent, with a fatality rate of 10.6%, compared to Type I, which has a fatality rate of 3.6%, as reviewed in (4).

The recent emergence of outbreaks in 2024 has intensified global concerns about their potential spread. This outbreak and the one in 2022 affected non-endemic countries, underscoring the pandemic potential of emerging orthopoxviruses (5, 6). As of August 23, 2024, 3,331 confirmed human cases of mpox and 17,389 suspected cases had been reported on the African continent. The outbreak has resulted in 582 deaths, indicating an estimated fatality rate of 2.7% (7). The 2022 outbreak accelerated research and development of vaccines, but related publications significantly declined in 2023. However, by 2024, the issue has regained critical importance.

Recent genomic changes in MPXV, including insertions and deletions, have been reported (2, 8). These changes occur in conserved regions common across orthopoxviruses and sections specific to MPXV strains. These alterations affect the functionality of critical genes, such as those related to immune modulation and viral replication, contributing to the virus’s genetic diversity. Such adaptations could enable the virus to infect new hosts or thrive in different ecological conditions, increasing its pandemic potential (9). This underscores the importance of continuous surveillance and the need to be prepared by reinforcing research and developing new vaccination strategies to address future epidemiological threats.

Recently, groundbreaking vaccine candidates against MPXV have been developed, including cutting-edge multivalent mRNA and DNA vaccines based on identifying immunogenic proteins from the MPXV capsid. This review will delve into the cutting-edge advancements in mpox vaccine production, providing a critical overview of these emerging technologies and highlighting the urgent need to push the boundaries of research and development to create vaccines that can offer strong immune responses, durable protection, a significant reduction in symptoms, effective neutralization of the virus, and comprehensive disease prevention, all while ensuring safety for global populations.

## 2 Pathophysiology of mpox

MPXV enters the human body mainly through skin wounds, mucous membranes, or the respiratory tract. After entry, the virus replicates at the entry site before spreading to regional lymph nodes, where a second replication occurs. The virus then spreads from the lymph nodes to other organs via the bloodstream, leading to viremia, which distributes the virus throughout the body. Viral replication triggers an immune response that includes inflammation and the release of cytokines and chemokines, contributing to systemic symptoms such as fever, muscle aches, and general discomfort. A distinctive feature of mpox compared to other orthopoxvirus infections is lymphadenopathy (inflammation of the lymph nodes), which appears early in the disease. Complications and clinical symptoms of mpox are more common in children and immunocompromised individuals. They can include pneumonia, respiratory failure, sepsis, encephalitis, and corneal infection, leading to vision loss, reviewed in (10, 11).

Once the patient is exposed to the virus, the incubation phase begins, typically lasting between 7 and 14 days, with a maximum of 21 days. During this period, the virus replicates and spreads to the lymph nodes. Initial viremia leads to viral expansion and colonization of other organs. It is suggested that during this stage, the host enzyme APOBEC3, a cytidine deaminase capable of converting cytosine to uracil in exogenous DNA, may induce changes in the virus’s genetic material. These incidental changes could contribute to the virus’s genetic diversity and the emergence of new variants, increasing its pandemic potential, as reviewed in (12).

During the secondary viremia, symptoms begin and last between 0 and 5 days. This stage, known as the prodromal phase, is characterized by fever, headache, lethargy, myalgia, and lymphadenopathy, followed by a rash that lasts 2 to 4 weeks. The skin rash progresses through several stages: macules, papules, vesicles, pustules, and finally, crusts. These lesions are usually concentrated on the face and extremities but can appear anywhere. During this phase, serum antibodies become detectable, and patients can be infectious. The infection is typically treated with antiviral drugs such as cidofovir, ribavirin, tecovirimat, and brincidofovir, inhibiting viral DNA replication and development (13).

Most mpox cases are self-limiting, but complications can occur, especially in individuals with compromised immune systems. These complications can include secondary skin infections, pneumonia, sepsis, and even death. Infection resolution typically happens with the gradual recovery and shedding of scabs, often leaving scars. Immunity developed after infection may protect against future reinfections, although the extent and duration of this protection are not fully understood (14).

**Figure 2:**
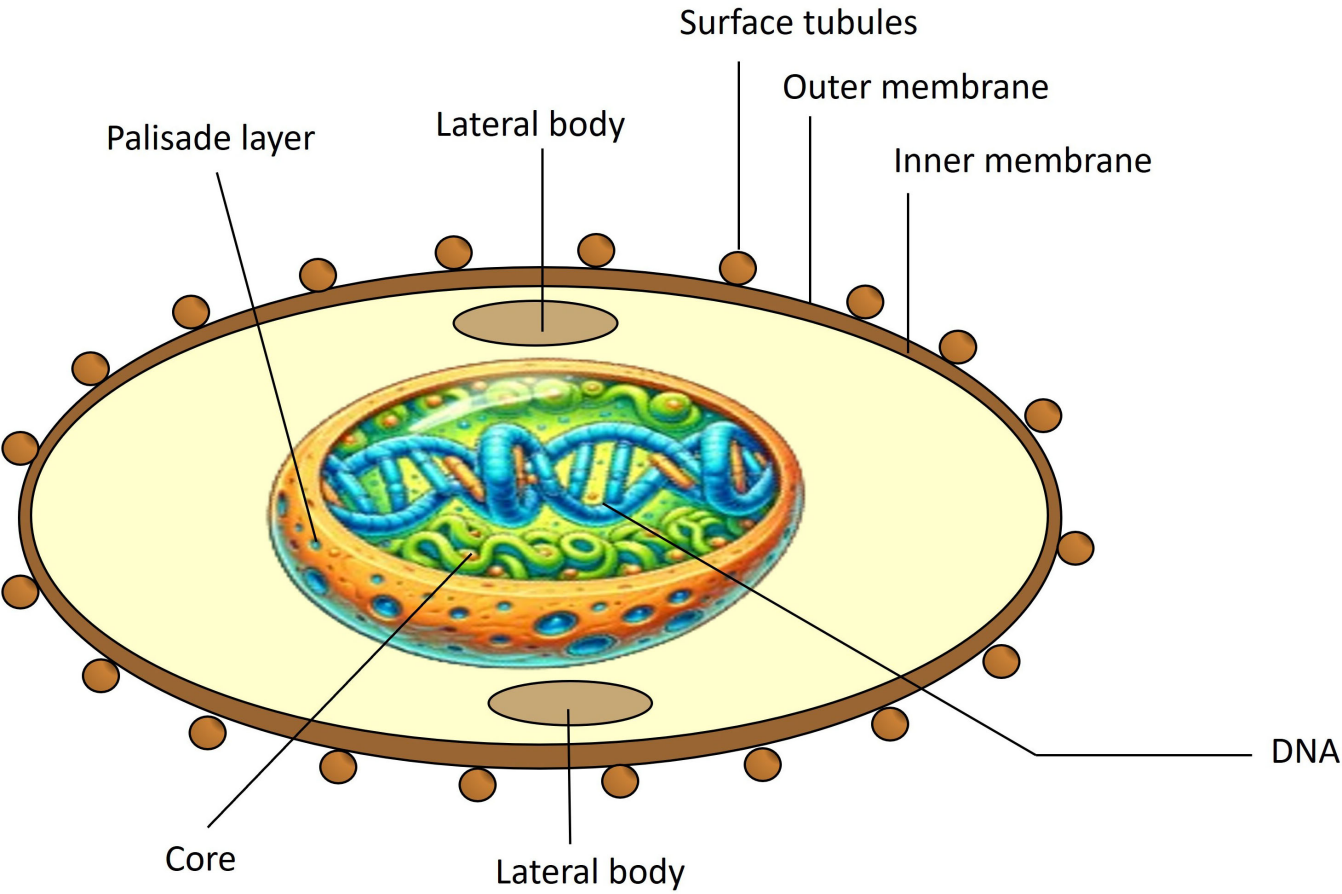
Structure of MPXV. MPXV consists of surface tubules, outer and inner envelopes of the extracellular virion, lateral bodies, a palisade layer (a protein layer in the core with parallel fibrils resembling a fence), and core fibrils.

MPXV viral particles are oblong, measuring 200 to 250 nm in diameter. The virion consists of five distinct structures: core, membrane, lateral body, surface tubules, and nucleocapsid. The core has a bilobed shape and is surrounded by a double lipoprotein layer. **Figure 2** shows the MPXV structure. The MPXV genome consists of double-stranded DNA comparable to smallpox. Its central region is 101 kb and shares 96.3% of the identity with the same Vaccinia Virus (VACV) area. The MPXV genome also has two variable terminal regions containing a 64 kb inverted terminal repeat (ITR) with some ORFs, hairpin loops, and short tandem repeats (15). It is 197 kb long and encodes at least 190 proteins, 30 of which are structural. Of the 190 proteins encoded by the MPXV genome, approximately 20 to 30 have been explored for potential use in vaccines. These proteins, particularly those located on the surface of the virion, such as A27L and B5R, have been studied due to their role in eliciting strong immune responses. These surface antigens are highly conserved across MPXV, VACV, and the smallpox virus, making them prime candidates for cross-protective vaccines. The conservation of these critical antigens across various orthopoxviruses enhances their potential utility in broad-spectrum vaccines, which could provide immunity against multiple poxviruses. Detailed studies have shown that these conserved antigens are crucial for the virus’s ability to infect host cells and are, therefore, integral to the design of effective vaccines (16).

Genomic differences between MPXV and VACV in genes coding for proteins that affect cytokines such as IL-1, tumor necrosis factor, and interferon can alter the host’s immune response, influencing the transmissibility and severity of infection. These genetic variations might explain why MPXV is not as fatal or transmissible as the smallpox virus but has the potential to become a more efficient human pathogen under certain circumstances, as reviewed in (17).

**Figure 3:**
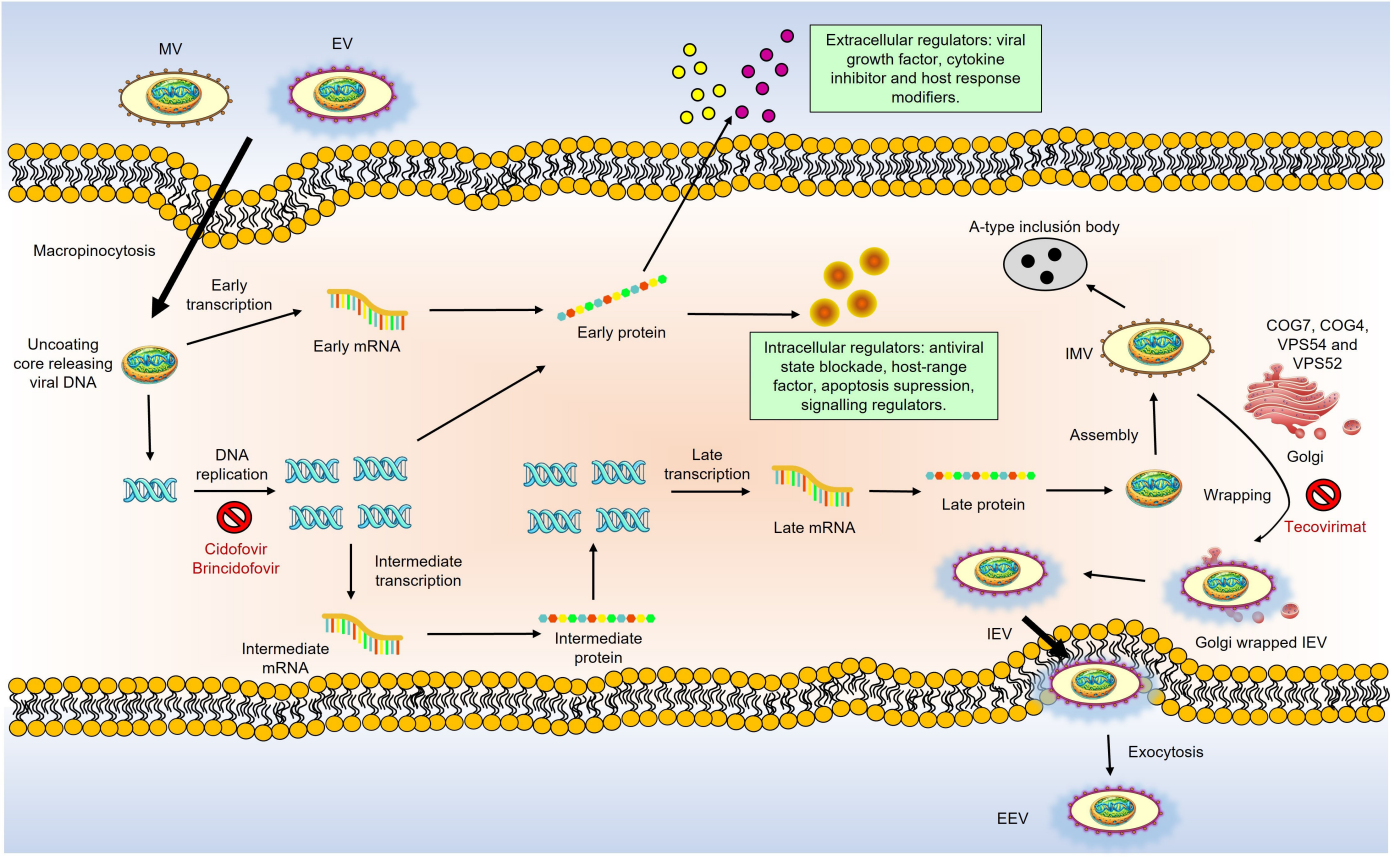
Schematic overview of MPXV life cycle and the action of anti-poxvirus drugs. MPXV replicates in the cytoplasm of infected cells. There are two infectious virions: intracellular mature virions (IMV) and extracellular enveloped virions (EEV), which differ in their membranes and glycoproteins. Virions bind to host cells via unknown receptors. MPXV replication involves early, intermediate, and late mRNA and protein synthesis stages, followed by virion assembly and morphogenesis. A double membrane from the Golgi wraps IMV to form intracellular enveloped viruses (IEV). GARP and COG complexes are crucial for infection, aiding in virus-cell interactions and the formation of EEVs. IEVs lose their outer membrane via actin-tail action, fuse with the cell membrane to become cell-associated enveloped viruses (CEVs) and are released as EEVs. MPXV also produces various modulatory proteins essential for replication. Anti-poxvirus drugs target different stages are pointed out in red: Cidofovir and brincidofovir inhibit viral DNA polymerase; tecovirimat and NIOCH-14 prevent CEV and EEV formation; Vaccinia Immune Globulin (VIG) prevents virions from infecting new cells.

Replication, assembly, and maturation of MPXV occur in the cell’s cytoplasm, as shown in **Figure 3**. Virus replication involves three stages: early, intermediate, and late synthesis of mRNA and viral proteins, followed by virion assembly and morphogenesis. Virion assembly takes place in the host cytoplasm. MPXV has two infectious variants: the Intracellular Mature Virion (IMV), which is infectious when released from damaged cells, and the Extracellular Enveloped Virion (EEV), which buds from infected cells. These particles differ in membrane layers and surface glycoproteins, are active in infection, and can cause disease, as reviewed in (18).

Similar to VACV, IMVs are believed to be the most infectious form of the virus, entering cells via macropinocytosis and mediating host-to-host viral transmission (19). During infection, intracellular enveloped viruses (IEVs) form in the Golgi, shed their outer membrane layers via actin-tail action, and fuse with the cell membrane to produce cell-associated enveloped viruses (CEVs). CEVs are released to form EEVs, which enter cells through membrane fusion and facilitate viral spread within the host (20).

The key receptors for viral entry into host cells have not yet been identified, but conserved oligomeric Golgi complexes, COG7 and COG4, are known to be involved in viral entry and fusion. The Golgi-associated retrograde protein (GARP) complex also plays a significant role in virus-cell interactions necessary for viral entry and exit. In contrast, proteins VPS52 and VPS54 are responsible for forming extracellular enveloped virions and MPXV infection, as reviewed in (21, 22). MPXV infection activates T-cells and stimulates the release of inflammatory mediators through receptor interactions. However, this effect is not observed in individuals with a prior history of mpox, indicating the virus suppresses T-cell activation (23). These findings show that the mechanisms involved in MPXV infection are not yet fully understood.

## 3 Current and clinical-stage of vaccines for MPXV

The development of vaccines against MPXV primarily leverages the immune response generated by the VACV. This approach is grounded in two key factors: the significant cross-protection between VACV and MPXV and the conservation of crucial viral proteins. Antibodies induced by first-generation smallpox vaccines can effectively neutralize MPXV, offering long-term protection. Studies have always shown that over 97% of tested samples-maintained neutralization titers above the protective level of 1:32, indicating that most vaccinated individuals retain significant neutralizing antibodies decades after their last smallpox vaccination. This long-lasting immunity primarily targets conserved envelope proteins of extracellular VACV, such as A33 and B5, and mature virion proteins, such as L1 and A27, also present on MPXV. These proteins are crucial for virus neutralization and immune protection in animal and human models. Because these antigens are conserved across the orthopoxvirus genus, they are targeted in current vaccine strategies to elicit broad immune protection against multiple orthopoxviruses, including MPXV (24, 25).

Currently, three vaccines are approved for MPXV worldwide: JYNNEOS, LC16m18, and ACAM2000. JYNNEOS, approved in the USA and Mexico, is a live, non-replicating vaccine produced using a modified Vaccinia Ankara virus, incapable of replicating in human cells. This makes JYNNEOS a safer option, particularly for immunocompromised individuals. A case-control study in the United States estimated the vaccine’s effectiveness as 36% after a single dose and 66% after two doses (26). Another study reported that the vaccination series provided 85.9% effectiveness (27). JYNNEOS is generally well-tolerated, with common side effects including mild injection site reactions and transient systemic symptoms such as fever and fatigue (28).

Another approved vaccine, LC16m8, is used in Japan. It is a live attenuated vaccine derived from the Vaccinia virus. It was specifically developed to enhance safety by removing virulence-associated genes. Clinical studies have demonstrated the immunogenicity of LC16m8, showing a seroconversion rate of neutralizing antibodies against MPXV by day 28 post-vaccination (29). The vaccine has a favorable safety profile, primarily mild and transient side effects like injection site reactions and mild systemic symptoms like headaches and fever (30).

ACAM2000, approved in the USA, is a live, replicating vaccine derived from the Vaccinia virus. Although initially developed for smallpox, it is also effective against MPXV due to the cross-protective immune response elicited by the Vaccinia virus. Historical data on smallpox vaccinations, which demonstrated cross-immunity to MPXV, support the use of ACAM2000. However, this vaccine is associated with more significant side effects compared to non-replicating vaccines, including myocarditis, pericarditis, and generalized VACV infection. As a result, its use is limited to non-pregnant, immunocompetent individuals (31), and its risks are well-documented (32).

When evaluating the available vaccines for MPXV, it is essential to balance immunogenicity and safety. ACAM2000, JYNNEOS, and LC16m8 offer different profiles of efficacy and side effects, which are crucial in shaping vaccination strategies against monkeypox and other orthopoxviruses. ACAM2000, based on a live attenuated Vaccinia virus, provides robust cross-protection against smallpox and monkeypox. However, due to its replicating virus, it carries higher risks of side effects, particularly in immunocompromised individuals. JYNNEOS, utilizing a non-replicating Modified Vaccinia Ankara (MVA) virus, offers a safer alternative, especially for vulnerable populations, though it may induce a weaker immune response. LC16m8, derived from a highly attenuated Vaccinia virus strain, balances safety and immunogenicity, showing reduced side effects while eliciting a robust immune response. However, its availability is primarily limited to Japan. A comparison between these three vaccines is shown in **Table 1**.

**Table 1:** Currently approved mpox vaccines.

Vaccine	Vaccine description	Approved country	Administration route and diagnosis	Recommendations for use	Side effects	Reference
ACAM2000	Attenuated VACV	USA	One-time dose via percutaneous route	Not recommended for immunocompromised individuals, pregnant or breastfeeding women, and individuals with cardiac risk. Recommended for individuals at high risk of contagion.	Myocarditis, pericarditis, primary erythema multiforme, encephalitis, progressive vaccinia, vaccinosis, eczema, blindness, and fetal death in pregnant women.	(108, 109)
JYNNEOS	Replication-deficient MVA vaccine	USA, Canada, Mexico, European Union	Two doses via SC.	Recommended for individuals at high risk of contagion.	Not reported in clinical trials.	(110, 111)
LC16m8	Attenuated, minimally replicating VACV	Japan	One-time dose via PC route.	Recommended for individuals at high risk of contagion.	Not reported in clinical trials.	(112)

A significant concern with attenuated virus vaccines like LC16m8 is the potential risk of reversion to a more virulent form. Although genetic modifications have been made to reduce this risk, there is no guarantee that the virus will not regain its pathogenicity under specific biological conditions (40). Additionally, attenuated viruses can recombine with endogenous viruses or other pathogens in the host, potentially creating new pathogenic viral forms that could evade immune control or existing treatments (33).

Non-replicating vaccines, such as JYNNEOS, also have their challenges, particularly in inducing a suboptimal immune response, especially regarding cellular immunity, which is crucial for protection against many viruses. This might necessitate multiple doses or the use of potent adjuvants to achieve the desired efficacy (34).

As of August 2024, the World Health Organization (WHO) recorded 22 clinical trials for MPXV vaccines, primarily based on attenuated or non-replicating viruses. These trials span Phases 1 through 4 and target diverse populations, including volunteers with no prior exposure to vaccinia, healthcare workers, men who have sex with men, and individuals living with HIV. Among the reviewed studies, only one (JPRN-jRCTs031220171) has reported results, while the remaining trials are either ongoing or recently completed. These studies aim to evaluate the safety, dosage, and efficacy of MPXV vaccines across various populations and contexts. It is worth highlighting a Phase I/II dose-escalation study is being conducted by BioNTech to assess the safety, tolerability, reactogenicity, and immunogenicity of the investigational RNA-based multivalent vaccine candidate BNT166a for active immunization against monkeypox (mpox) (NCT05988203). This trial consists of two substudies: Substudy A (SSA), involving approximately 48 vaccinia-naïve participants receiving two doses of BNT166a 31 days apart across three dose levels, and Substudy B (SSB), with 16 participants who have a prior history of smallpox vaccination (vaccinia-experienced), following the same dosing schedule. The trial began with a sentinel group in SSA, followed by dose escalation and expansion cohorts. While the initial design included a second candidate, BNT166c (trivalent), this arm was not activated. **Table 2** summarizes the essential details of these clinical trials, including vaccine type, trial phase, vaccination scheme, target populations, and the availability of results.

**Table 2:** Mpox vaccines in WHO-Registered clinical trials.

Trial registration	Vaccine	Vaccination scheme	Phase	Population	Results available
JPRN-jRCTs031220171	LC16m8	Vaccine administered to all participants.	1	Volunteers without previous exposure to vaccinia (n=50).	(29)
NCT05762523	VACΔ6	Group 1: a single dose of 10^6^ PFU; Group 2: a single dose of 10^7^ PFU; Group 3: two doses of 10^6^ PFU administered 28 days apart; Group 4: one dose of smallpox vaccine followed by a second dose of 10^6^ PFU 7 days late	1	Volunteers without previous exposure to vaccinia (n=60).	No
NCT05988203	BNT166a	Substudy A involves a dose-escalation study across three dose levels in participants without a prior smallpox vaccination, administering two doses 31 days apart. Substudy B focuses on individuals with a history of smallpox vaccination, also receiving two doses 31 days apart.	1 and 2	VACV-naïve volunteers and VACV-experienced	No
NCT05995275	mRNA-1769	The study involved four groups, each receiving a different dose, with vaccinations administered on days 1 and 29.	1 and 2	VACV-naïve volunteers	No
NCT05512949	JYNNEOS	Group 1: 2x10^7^ PFU; Group 2: 1x10^7^ PFU; Group 3: 1x10^8^ PFU.	2	Volunteers without previous exposure to vaccinia (n=229).	No
NCT05740982	JYNNEOS	Group 1 receives two doses, each a fifth of the standard MVA-BN (ID) dose; Group 2 receives two doses, each a tenth of the standard MVA-BN (ID) dose; Group 3 receives two standard doses of MVA-BN; Group 4 comprises adults following the Group 3 regimen; Group 5 consists of adolescents following the Group 3 regimen.	2	Volunteers without previous exposure to vaccinia (n=400).	No
JPRN-jRCTs031220137	LC16m8	A standard dose of the LC16m8 Smallpox Vaccine was administered following exposure.	2	Individuals without prior exposure to vaccinia who have been in close contact with mpox cases have not contracted the disease (n=150).	No
NCT06549530	MVA-BN	Two doses of the MVA-BN vaccine were administered with a 4-week interval between them.	2	Non-vaccinated	No
NCT05846243	VACΔ6 vaccine	Diverse vaccination doses were administered according to varying schedules.	2 and 3	Individuals receiving various vaccination schedules (n=334).	No
NCT02977715	JYNNEOS	Two SC vaccinations were administered four weeks apart from three different lots of MVA-BN.	3	Adult healthcare personnel at risk for mpox in the Democratic Republic of the Congo (n=1600).	No
DRKS00029638	JYNNEOS	The vaccination group was administered either one or two doses of the MVA-BN vaccine.	3	Men who engage in sexual activities with other men (n=15000).	No
NCT06223919	LC16m8 Smallpox Vaccine	Compare vaccination administered immediately versus vaccination given after a delay.	3 and 4	HIV-infected individuals with CD4 >200 µL, those on Prep, MSM	No
NCT05745987	JYNNEOS	Comparison between a single dose of MVA-BN and a single dose of TYPHIM Vi^®^ typhoid vaccine.	4	Individuals without prior exposure to vaccinia who have been in close contact with mpox cases within the last 14 days (n=1560).	No
NCT05734508	JYNNEOS	Two doses of the MVA-BN vaccine were administered four weeks apart.	4	Adult personnel and staff involved in the PALM-007 study in the Democratic Republic of the Congo (n=500).	No
RBR-10mpz6sd	JYNNEOS	Vaccinated and unvaccinated participants.	4	Individuals with prior exposure to mpox (n=746).	No
NCT03745131	JYNNEOS	Comparison of individuals before and following exposure and those who were not vaccinated.	N/A	Healthcare workers who were administered the MVA-BN vaccine during the mpox outbreak (n=120).	No
NCT05654883	JYNNEOS	Ten variations of SC and ID vaccinations were administered using fractional or standard doses, single doses, or boosters.	N/A	HIV-positive and HIV-negative individuals who received either fractional intradermal doses or standard doses of a vaccine against mpox (n=300).	No
NCT05562323	JYNNEOS	HIV-positive versus HIV-negative.	N/A	HIV-positive and HIV-negative individuals who received pre-exposure prophylaxis vaccination against mpox (n=100).	No
NCT05522296	Mpox vaccine	Vaccinated or unvaccinated against mpox and smallpox.	N/A	Individuals vaccinated and not vaccinated against mpox/smallpox (n=4638).	No
NCT05438953	IMVANEX and JYNNEOS	Two doses of the MVA vaccine were administered 28 days apart.	N/A	Unvaccinated (n=300).	No
NCT05627713	JYNNEOS	Vaccinated with the MVA-BN vaccine or suspected to be infected with MPXV.	N/A	Individuals eligible for vaccination or suspected of having MPXV infection (n=330).	No
NCT05879965	Smallpox Vaccine	MPXV infection compared to smallpox vaccination versus unvaccinated individuals with no history of mpox.	N/A	Individuals vaccinated with the smallpox vaccine or diagnosed with MPXV through PCR testing (n=345).	No

MVA-BN, Bavarian Nordic developed MVA under the brand name MVA-BN^®^, ID intradermal; SC is subcutaneous.

N/A, Not Applicable.

The analysis of vaccine platforms used in mpox development reveals distinct advantages and limitations across different technologies. Attenuated virus vaccines provide broad immune activation but carry risks such as reversion to virulence and require a cold chain. Non-replicating vaccines are safer for immunocompromised individuals but often elicit suboptimal immune responses. Viral vector-based vaccines offer durable immunity and can be developed rapidly, though they face challenges such as pre-existing immunity and complex manufacturing processes. Recombinant vaccines are safe and easy to store but may have lower immunogenicity without adjuvants. DNA vaccines are quickly developed and stable at room temperature but require advanced delivery systems. Lastly, mRNA vaccines are highly scalable and non-integrative but are associated with inflammatory responses and require an extreme cold chain for storage. Each platform must balance these trade-offs to optimize efficacy, safety, and logistical feasibility during vaccine development and deployment. **Table 3** provides a comparative analysis of different vaccine platforms, highlighting their advantages and limitations.

**Table 3:** Comparison of different platforms used in mpox vaccine development.

Vaccine platform	Advantages	Limitations
Attenuated Virus Vaccines	• Broad immune activation. • Established technology.	• Risk of reversion to virulence. • Recombination with endogenous viruses. • Requirement for cold chain.
Non-Replicating Vaccines	• Minimal risk of reversion. • Suitable for immunocompromised individuals. • Established technology.	• Suboptimal immune response. • Limited efficacy in immunocompromised individuals. • Requirement for cold chain.
Viral Vector-Based Vaccines	• Durable immunity. • Flexibility and rapid development. • No need for adjuvants. • Single dose efficacy.	• Pre-existing immunity. • Complex manufacturing. • Potential for recombination. • Limited use in immune-compromised populations. • Requirement for cold chain.
Recombinant Vaccines	• High safety profile. • Strong immune response. • Stable and easy to store. • No risk of genetic integration.	• Lower immunogenicity without adjuvants. • Complex and costly production. • Potential for allergic reactions. • Limited cell-mediated immunity. • Requirement for cold chain.
DNA Vaccines	• Rapid development and production. • Stability at room temperature. • Versatility in design.	• Lower immunogenicity. • Potential for genomic integration. • Need for Advanced Delivery Systems. • Requirement for cold chain.
mRNA Vaccines	• Efficient protein expression. • Rapid scalability. • Non-Integrative. • Customizable and Adaptive.	• Inflammatory Nature. • Poor stability and delivery challenges. • Requirement for cold chain. • Need for Advanced Delivery Systems. • Requirement for extreme cold chain.

### 3.1 Vaccines based on attenuated or non-replicating viruses

New vaccine candidates against MPXV, based on attenuated or non-replicating viruses, are currently in preclinical stages. These vaccines introduce a safer form of the virus, which cannot cause disease but is sufficient to stimulate an immune response. Attenuated viruses are less virulent versions, while non-replicating viruses are designed not to replicate within the host. **Table 4** discusses these preclinical vaccine candidates and reveals a variety of approaches, models, and outcomes.

**Table 4:** List of preclinical mpox vaccine candidates based on attenuated and non-replicating viruses.

Vaccine description	Biological Model	Vaccination scheme	Challenge	Main results	Reference
Attenuated virus NYCBHΔE3L with the E3L gene deleted.	Cynomolgous monkeys	Group 1: vaccinated with NYCBHΔE3L days 0 and 21. Group 2: NYCBH on day 21. Group 3: mock vaccination.	Mpox vaccine on day 49 since first vaccination.	All NYCBH-vaccinated animals and most NYCBH E3L-vaccinated ones survived MPXV, unlike all mock-vaccinated animals who succumbed. NYCBH animals had fewer lesions; those with NYCBH E3L showed many lesions initially, but these healed by day 21.	(35)
MVA	Cynomolgous monkeys	Group 1: intramuscular inoculation with 10^8^ PFU of MVA at zero time and a second two months later; Group 2: one IM injection with 10^8^ PFU of MVA followed two months later by a standard percutaneous inoculation with Dryvax; Group 3: nothing at zero time and one Dryvax inoculation two months later; Group 4: unimmunized control.	Mpox virus strain zaire 79 two months after last vaccination	Vaccinated monkeys with MVA or Dryvax didn’t develop lesions, unlike those who did. A couple of MVA-vaccinated monkeys had a single lesion compared to the 11-36 in their unvaccinated counterparts. Vaccination also led to lower viremia levels, confirmed by PCR.	(36)
Recombinant MVA	Rhesus macaques	The animals received two doses of recombinant MVA, each with 2×10^8^ infectious units, one month apart. The vaccines were administered via IM, ID, and Palatine Tonsil.	IV MPXV challenges 2 and half years after the 2nd MVA/KB9-5.	Vaccinated animals had significantly lower viral loads than unvaccinated ones, with clear differences observed for over a week. Unvaccinated monkeys experienced severe symptoms and many lesions, which healed in about three weeks, whereas vaccinated monkeys had minimal, less severe lesions that healed quickly without showing illness. Also, the vaccine’s effectiveness did not correlate with the intensity of initial immune responses.	(113)
MVA or Dryvax	Cynomolgous monkeys	MVA was administered via IM at a dose of 10^8^ infectious units per macaque. Dryvax was given via ID at a dose of 2.5×10^5^ infectious units per macaque.	Mpox virus IV 5x10^7^ and 5x10^6^	High-dose challenged naive animals developed lesions within a week and were euthanized. In a moderate-dose group, lesions appeared by day 6 but resolved in most animals. Vaccinated animals had much lower viremia levels than unvaccinated ones. MVA vaccine led to quicker antibody responses than Dryvax.	(114)
Dryvax and ST-246	BALB/c Mice	Mice were administered the Dryvax smallpox vaccine and ST-246. Each mouse received 5×10^5^ PFU of Dryvax delivered dermally at the base of the tail with subsequent scarification. Alongside, mice were given 2 mg of ST-246 orally each day at 24-hour intervals, starting on the vaccination day, for 7 or 14 consecutive days.	A lethal dose of VACV Western Reserve (WR).	The vaccine with ST-246 maintained cellular and neutralizing antibody responses, although anti-vaccinia ELISA titers were slightly reduced. Combined with the vaccine, ST-246 offered comparable protection against the lethal intranasal VV-WR challenge to the vaccine alone.	(115)
JYNNEOS or ACAM2000.	Cynomolgous monkeys	JYNNEOS: Administered as a single dose of 1x10^8 TCID50 via SC 28 days before the challenge. A prime-boost regimen included a primer dose given 56 days before and a booster dose 28 days before the challenge. ACAM2000: It is administered as a single dose, ranging from 2.5x10^5^ to 12.5x10^5^ PFU, by scarification 28 days before the challenge.	Mpox virus strain Zaire 79 (NR-2324).	Neutralizing antibodies were similar between animals given a single Acam2000 dose (132 U/ml) and those on the JYNNEOS prime-boost regimen (69 U/ml) before monkeypox exposure. Post-challenge, 2 of 6 JYNNEOS animals showed viral excretion, none in the ACAM2000 group.	(116)
LC16m8 and Lister (Elstree) strain.	Cynomolgous monkeys	Single dose of LC16m8, 1×10^8^ PFU/mL. at two intervals post-vaccination: 1) 6 months after vaccination with LC16m8 for one group of monkeys, 2) 12 months after vaccination with LC16m8 for another group of monkeys.	MPXV strain Zr-599	LC16m8 monkeys exhibited few monkeypox symptoms, unlike most naïve monkeys, which died. LC16m8 triggered a protective immune response, evidenced by quick viremia reduction and IgG antibody reaction.	(117)
Dryvax, ACAM2000, or JYNNEOS	Prairie dog (*Cynomys ludovicianus)*	Dryvax and ACAM2000 vaccines with a single dose of 2×10^5^ PFU 28 days before the challenge. JYNNEOS was given in two protocols: a single dose of 1× 10^8^ TCID50 28 days before the challenge, a two-dose regimen with the first dose 60 days prior, and a booster 30 days before the challenge.	10^5^ or 10^6^ PFU Congo Basin MPXV 30 and 60 days after vaccination.	Vaccination with Dryvax or Acam2000 prevented death and rash in animals. JYNNEOS also prevented death but caused a modified rash. All vaccines produced antiOPXV IgG antibodies.	(118)
JYNNEOS or ACAM2000	Prairie dog (*Cynomys ludovicianus*)	Vaccines were given either 1 day or 3 days post-exposure. JYNNEOS: Administered at a dose of 1×10^8^ TCID per animal. ACAM2000: Administered at a dose of 2×10^5^ PFU per animal.	Mpox virus at doses of 10^4^ PFU (2xLD_50_) or 10^6^ PFU (170LD50)	JYNNEOS vaccination on Day 1 post-exposure had the lowest mortality rate at 12% (1/8). Higher mortality rates were noted with later or different treatments: 62% (5/8) with JYNNEOS on Day 3, 50% (4/8) with ACAM2000 on Day 1, and 38% (3/8) with ACAM2000 on Day 3. There were no significant differences in lesion counts between groups, though vaccinated animals had lower counts, developed neutralizing antibodies, and controlled viremia post-challenge.	(119)
Live smallpox vaccine	BALB/c Mice	The Lister vaccine was administered to mice via scarification or IM injection, using up to 1x10^7^ PFU.	10^7^, 10^8^, or 3.4X10^8^ PFU of the VACV strain WR, measured 28 days after vaccination.	No significant differences were found in clinical scores between IM and scarification routes at any dose (P ≥ 0.13); both protected effectively against severe disease and death.	(120)
ACAM2000 and the antiviral Tecovirimat	Cynomolgous monkeys	ACAM2000 was administered with either tecovirimat or a placebo. The dose was 1.0–5.0x10^8^ PFU/mL, or 2.5–12.5x10^5^ PFU per dose, delivered into the intrascapular region by percutaneous scarification with bifurcated needles. Tecovirimat was given concurrently at 10 mg/kg daily for 14 days. Post-vaccination, monkeys faced a lethal monkeypox virus challenge 28 to 45 days later, varying by study.	MPXV 5.0x10^7^ IV either at day 30, 32, or 45 post-vaccination.	7/13 ACAM2000 + tecovirimat animals had positive vaccine responses. Antibody levels were higher in the ACAM2000 + placebo group. Post-lethal MPXV challenge, all placebo-treated animals survived (12/12), and 12/13 tecovirimat-treated animals did too. Tecovirimat-treated animals exhibited more severe clinical signs than those treated with a placebo.	(121)
Dryvax	Cynomolgous monkeys	Dryvax: specific dosage details were not provided in the text excerpt. Cidofovir: It was administered with Dryvax to control active VACV infection, 0.5 mg/kg, once daily, starting on the day of vaccination and continuing for 14 consecutive days.	MPXV two months post-vaccination.	Dryvax vaccination fully protected against lethal monkeypox challenges, triggering gamma interferon production in activated Vγ2Vδ2 T-cells. Even when suboptimally primed, these T-cells in co-vaccinated macaques significantly expanded after the challenge, accumulating in inflamed lung tissues. No viral mRNA was detected from day 4 to 27 post-challenge.	(122)

MVA, Modified Vaccinia Ankara; IM, intramuscular; ID, intradermal; antiOPXV, antibodies that target orthopoxviruses; PFU, plaque-forming units; SC, subcutaneous; Dryvax, a live-virus preparation of VACV prepared from calf lymph; ST-246, is a poxvirus dissemination inhibitor; TCID, tissue-culture infectious dose.

For instance, attenuated virus vaccines like NYCBHΔE3L, tested on cynomolgus monkeys, showed strong survival rates post-MPXV challenge, with fewer lesions than unvaccinated controls. Similarly, recombinant MVA vaccines showed efficacy in rhesus macaques by significantly lowering viral loads and resulting in minimal, quickly healing lesions (35).

In addition, Dryvax and ST-246, when administered to mice, maintained immune responses and protected lethal challenges, similar to the JYNNEOS and ACAM2000 vaccines tested in both cynomolgus monkeys and prairie dogs. These vaccines successfully induced neutralizing antibodies and controlled viremia, with some variations in post-exposure effectiveness depending on the timing of administration. Furthermore, live smallpox vaccines protected BALB/c mice against severe disease and death, regardless of the administration route. In cynomolgus monkeys, the combination of ACAM2000 with the antiviral Tecovirimat showed high survival rates after a lethal challenge, although tecovirimat-treated animals exhibited more severe clinical signs. Dryvax and Cidofovir also demonstrated full protection against MPXV in monkeys, highlighting a significant expansion of activated T-cells post-challenge, indicating a strong immune response (36).

The development of attenuated and non-replicating virus-based vaccines for mpox has made significant progress, notably with vaccines like ACAM2000 and JYNNEOS. ACAM2000 is effective but can cause serious side effects, including cardiac issues, as reviewed in (37). JYNNEOS, a non-replicating vaccine, has fewer complications, offering a safer and more effective option for mpox prevention (38). These vaccines, initially developed for smallpox, are identical in formulation but have been applied to mpox, demonstrating their effectiveness against both diseases and highlighting the adaptability of this approach for combating emerging viral threats.

Live virus-based vaccines have strengths and limitations. Their production is based on well-established technology, and they currently represent the majority of vaccines undergoing clinical evaluation. However, other types, such as mRNA vaccines, are also being tested, including BioNTech’s Phase 1 clinical trial (ClinicalTrials.Gov ID NCT05988203) (39). These vaccines can induce a robust immune response similar to a natural infection, effectively stimulating cellular and humoral immunity. However, drawbacks include the potential risk of reversion to virulent forms in attenuated viruses and the need for adjuvants or complex delivery systems for non-replicating viruses to be effective (40). Additionally, large-scale production of live virus vaccines is technically challenging and costly, requiring specialized biosafety facilities that comply with strict regulations to ensure safety, efficacy, and quality control. Facilities must adhere to Good Manufacturing Practices (GMP), which, besides being expensive, are crucial for controlling contamination and ensuring product consistency (41).

Given the risks associated with live virus vaccines, further research is essential to develop safer and more cost-effective vaccination platforms that facilitate universal access. The following sections will explore some of these emerging platforms in detail.

### 3.2 Viral vector-based vaccines

Viral vector-based vaccines that do not use live viruses offer multiple benefits. They utilize non-replicating viruses, minimizing the risk of disease, which is particularly important for people with weakened immune systems. These vaccines can be designed to express specific antigens, generating a robust immune response without the risks associated with the use of live pathogens. This also allows for rapid adaptation to emerging pathogens. Additionally, they induce both humoral and cellular immune responses, protecting against viral infections (42, 43).

However, while viral vector-based vaccines can induce strong immune responses, the spectrum of the immune response may be limited. Depending on the vector and antigen used, some vectors may be more effective inducing a humoral reaction but less effective in generating a cellular response, or vice versa. One major challenge is preexisting immunity in the population. Many people may have preexisting immunity to the viral vector (such as adenoviruses), which can neutralize it before the viral antigen delivery, reducing its effectiveness (44). Additionally, the production and storage of these vaccines can be more complex due to the need to handle viruses.

The development of viral vector-based mpox vaccines has shown advantages inducing robust and long-lasting immune responses. Novel vaccine design technologies, such as reverse vaccinology and immunoinformatics, facilitate identifying and optimizing immunogenic epitopes derived from MPXV proteins. **Table 5** shows some preclinical trials of viral vector-based vaccine candidates against mpox.

**Table 5:** Preclinical viral vector-based mpox vaccine candidates.

Vaccine description	Biological Model	Vaccination scheme	Challenge	Main results	Reference
BoHV-4, expressing MPXV antigens A29L, M1R and B6R	Mice 129 STAT1(-/-)	BoHV-4s vectors were administered via IP and MVA at 2x10^8^ PFU/mL via SC. Primary vaccination was given on day 0, and a booster was given on day 23.	2x10^5^ PFU of MPXV, 50 days after the initial vaccination.	The research demonstrated that recombinant BoHV-4 vectors expressing specific MPXV glycoproteins (A29L, M1R, and B6R) could protect STAT1 knockout mice from a lethal MPXV challenge. Among the tested vectors, the one expressing the M1R glycoprotein provided the highest level of protection, especially when administered as a prime-boost regimen.	(45)
Chimeric Recombinant Horsepox Virus (TNX-801).	Cynomolgous monkeys	On Day 0, animals received a single dose of the TNX-801 vaccine, either a high dose at 1.58×10^6^ PFU or a low dose at 2.51×10^5^ PFU. The vaccine was administered through scarification.	The challenge involved administering a lethal dose of MPXV strain Zaire via intratracheal inoculation.	The vaccine protects against the lethal MPXV Zaire strain, preventing severe animal disease and lesions.	(46)
Recombinant adenovirus encoding VACV A27L glycoprotein.	Mice	Mice were immunized with a single IM injection of VACV A27L. Each mouse received 0.5x10^9^ PFU injected into each hind leg.	The challenge involved administering a lethal IN dose of the VV-WR strain to the mice, five times the LD50.	Significant reductions in post-challenge morbidity correlated with strong neutralizing antibody and polyfunctional T-cell responses. The rAd-A27L vaccine’s effectiveness lasted at least 35 weeks post-vaccination.	(47)
Non-replicating vaccinia virus (NTV), derived from the Tian Tan strain	Mice, Rhesus Monkey	One group of mice (n = 7) was administered 2 × 10^5^ PFU of replicating VACV VTT through skin scratches, while another group (n = 7) received 2 × 10^7^ PFU of NTV via intramuscular injection. Likewise, one group of monkeys (n = 4) was inoculated with 10^6^ PFU of VTT through skin scratches, and the other group (n = 4) received 10^8^ PFU of NTV by intramuscular injection.	The animals were challenged with 2 × 10^5^ PFU of replicating VACV for the VTT group via skin scratches and 2 × 10^7^ PFU for the NTV group via intramuscular injection​.	The non-replicating VACV NTV vaccine proved to be as effective as the replicating VACV VTT in generating neutralizing antibodies against both mpox and VACV in mouse and rhesus monkey models.	(123)

BoHV-4, Bovine Herpesvirus 4; IP, intraperitoneal; SC, subcutaneous; IM, intramuscular; IN, intranasal; VW-WR, Vaccinia virus Western Reserve; LD_50_, Lethal dose 50.

Comparing vaccine technologies across various studies reveals diverse approaches, biological models, vaccination schemes, and outcomes. One of the vaccines, the BoHV-4 Expressing MPXV Antigens vaccine, was tested in mice using intraperitoneal (IP) and subcutaneous (SC) vaccination routes. Results indicated that mice vaccinated with MVA were fully protected against the MPXV challenge, underscoring the critical role of the M1R antigen in achieving protection. However, the combination group (Combo/Combo) exhibited slightly lower protection rates, suggesting that a booster dose is essential for optimal efficacy (45). The Chimeric Recombinant Horsepox Virus (TNX-801), tested in cynomolgus monkeys, provided robust protection against a lethal MPXV Zaire strain, preventing severe disease and lesions (46). The Recombinant Adenovirus encoding VACV A27L Glycoprotein, tested in mice, significantly reduced morbidity after a lethal challenge, with sustained effectiveness for at least 35 weeks, indicating strong and long-lasting immunity (47).

Modified Vaccinia Ankara (MVA) vaccines, evaluated in multiple studies, consistently demonstrated reduced lesion development and lower viremia levels in vaccinated monkeys. MVA stands out as an effective platform for vaccine development. These vectors can deliver multiple selected epitopes that generate cellular and humoral immunity. Improving the immunogenicity of MVA-vectored vaccines has been a focal point in recent research, mainly through optimizing vector design and promoter utilization. One strategy involves deleting specific genes that modulate the innate immune response, which has been shown to enhance the quality and specificity of T-cell responses. For instance, the simultaneous deletion of the A44L, A46R, and C12L genes from the MVA genome resulted in significantly improved adaptive and memory T-cell responses, highlighting the potential for more effective vaccine vectors (48). Additionally, the use of stronger endogenous promoters, such as pF11 and pB8, has demonstrated a notable increase in transgene expression and overall immunogenicity, offering a promising avenue to enhance the efficacy of MVA-based vaccines, particularly in the context of emerging infectious diseases like mpox (49). These vaccines provide a robust safety profile and the ability to induce strong humoral and cellular immune responses, making them highly advantageous for use in diverse immunization strategies.

Building on these advancements, reverse vaccinology offers an innovative approach to refine vaccine design further. This method allows for incorporating T-cell and B-cell epitopes identified through immunoinformatics tools, enhancing vaccine specificity and effectiveness. By targeting critical immune components, reverse vaccinology complements the work done with MVA and other platforms, ensuring that vaccines elicit robust CD4+ and CD8+ T-cell responses, crucial for recognizing and eliminating infected cells. Additionally, potent B-cell responses lead to the production of neutralizing antibodies, providing long-term immunity. For instance, Bhattacharya et al. (2022) utilized immunoinformatics methods to design a vaccine targeting multiple virus epitopes, optimizing its ability to induce a solid and comprehensive immune response (50). Current vaccine strategies, including those for viral vector-based vaccines, have demonstrated both T-cell and B-cell mediated responses, contributing to their protective efficacy. While this reverse vaccinology approach has not yet been explicitly tested in viral vector-based vaccines, the principles of epitope design could be applied to enhance the effectiveness of such vaccines. Similarly, Ullah et al. (51) demonstrated how immunoinformatics analyses can predict vaccine constructs’ stability, immunogenicity, and non-allergenicity, which could also be leveraged to improve the formulation of viral vector-based vaccines (51).

Integrating these approaches accelerates vaccine development and creates safer and more effective vaccines for combating monkeypox and other emerging pathogens. Combining these innovative methods promises to transform the vaccine landscape as we progress, offering more adaptive and targeted strategies to combat viral outbreaks.

### 3.3 Recombinant protein-based vaccines

Recombinant protein-based vaccines utilize specific pieces of a pathogen, such as proteins, produced through genetic engineering techniques. These proteins are recognized by the immune system as foreign, triggering an immune response without the need for live or inactivated infectious agents, as reviewed in (52). Since they do not use the complete pathogen but only specific parts, they have a higher safety profile, with no risk of reactivation or causing disease. The most promising vaccine candidates are designed to induce strong immune responses by presenting viral antigens in a way that engages Toll-like receptors (TLRs) and the major histocompatibility complex (MHC), both of which are crucial for effective immune system activation. TLRs recognize pathogen-associated molecular patterns (PAMPs), including viral antigens or their molecular components. By incorporating adjuvants or specific viral components that mimic these PAMPs, the vaccine formulation ensures that the viral antigens are detected by TLRs, thereby triggering an innate immune response. This initial activation facilitates the presentation of antigens by MHC molecules to T cells, further amplifying the adaptive immune response. Additionally, they have good stability and do not require special storage conditions like live attenuated vaccines, as reviewed in (53).

The development of recombinant protein-based vaccines against mpox is progressing, with several studies evaluating their efficacy and safety. Current approaches include using specific viral proteins that induce protective immune responses. Preclinical studies on recombinant protein-based vaccines against monkeypox are shown in **Table 6**. Comparing vaccine candidates in various studies reveals diverse approaches, biological models, vaccination schemes, and outcomes.

**Table 6:** List of Preclinical Recombinant Protein-Based Mpox Vaccine Candidates.

Vaccine description	Model	Vaccination scheme	Challenge	Main results	Reference
A subunit smallpox vaccine with VACV proteins A33, B5, L1, A27, and CpG/alum.	Cynomolgous monkeys	Group 1 received three doses of 100 µg of each protein with CpG/alum adjuvants at weeks 0, 4, and 12. Group 2 received two doses at weeks 0 and 4.	The 3-dose group received a lethal MPXV dose 5 weeks after the final dose; the 2-dose group 4 weeks later.	All animals in the three-dose group survived; one in the two-dose group did not. Disease signs were the least frequent in the three-dose group. The Dryvax group had higher antibody levels than controls. Tetravalent vaccines with CpG/alum enhanced neutralization titers and significantly reduced viral loads compared to controls.	(54)
Recombinant subunit smallpox vaccine with A27V, A33V, B5V, and two adjuvants: aluminum hydroxide and CpG.	Mice BALB/c	Mice were vaccinated with 2 µg or 10 µg doses containing proteins in histidine buffer and Alhydrogel, with optional CpG adjuvant, and boosted two weeks later.	Intranasal VACV (1.7×10^5^ PFU).	Groups vaccinated with adsorbed/non-adsorbed L1V and adsorbed B5V had minimal weight loss and high survival rates (80-100%). Other groups, like adsorbed A33V (100% survival), non-adsorbed A33V (80%), and non-adsorbed B5V (60%), lost more weight (about 20%). A27V formulations elicited strong antibody responses but failed to protect against virus infection effectively, mirroring results with the histidine-tagged VACV A27 protein.	(55)
A polyvalent vaccine	Mice BALB/c	Animals were immunized with a recombinant protein vaccine comprising MPXV proteins A29L, M1R, A35R, and B6R, combined with 15 mg of QS-21 adjuvant via SC on a three-dose regimen: the initial dose on day 0, followed by boosters on days 21 and 42.	A lethal dose of the MPXV strain WIBP-MPXV-001 was administered to BALB/c mice. The viral dose used for the challenge was 2.24x10^8^ PFU/mL, and it was given via IN and IP after the final immunization.	After the initial boost, antibody levels in mouse sera increased significantly, enhancing IFN-γ production and Th1-mediated cellular immunity. The vaccine also notably reduced MPXV replication and organ damage in mice.	(56)
Escherichia coli-Expressed VACV A27L, B5R, and D8L.	Mice BALB/c	BALB/c mice were immunized with Escherichia coli-derived A27L, D8L, and B5R proteins using either monophosphoryl lipid A and trehalose dicorynomycolate adjuvant or TiterMax Gold adjuvant.	Mice were challenged via IN with 20 times the 50% lethal dose (LD_50_) of VV-WR, amounting to approximately 2.4x10^6^ PFU.	Three immunizations with A27L, D8L, and B5R, or just A27L and B5R, produced strong antibodies and fully protected mice against a lethal dose of VACV. Recombinant proteins neutralized the virus and protected it from lethal challenges, and protein immune serum also offered protection.	(57)
MPXV-B6R antigen was combined with BC02, a new adjuvant developed using proprietary technology.	Female BALB/c mice.	The vaccination schedule in this paper involved two intramuscular immunizations with 5 µg of antigen combined with adjuvant BC02. The doses were administered at one-week and three-week intervals, and the immune response was tested four weeks after the final immunization.	The mice were challenged by administering two intramuscular vaccine doses at one- and three-week intervals. Four weeks after the final immunization, the immune response of the mice was tested. Additionally, the mice were exposed to the VACV to measure neutralizing antibody titers and assess the protective efficacy of the immunization​​.	A single dose of B6R-BC02 triggered a faster humoral immune response than naked B6R, with serum IgG2a levels rising rapidly. BC02 enhanced memory immune responses and showed strong, quick responses when antibody levels dropped, boosting overall immunity. Similar effects were observed in memory B-cell and T-cell responses.	(124)
MPXV protective antigens, L1, A29, A33, and the thermostable Aquafex aeolicus lumazine synthase (AaLS) were expressed in E. coli.	BALB/c mice	Three intramuscular doses of the nanovaccine to the mice at weeks 0, 2, and 4. Each dose contained 5 µg of antigen.	The animals were challenged with the vaccinia virus after receiving two doses of 20 µg of the mRNA vaccine at 2-week intervals. Thirty days after the first vaccination, they were exposed to 1x10^6^ PFU of the virus intranasally​.	The study developed thermostable nanovaccine candidates by conjugating MPXV antigens with the AaLS scaffold. These vaccines showed high stability at elevated temperatures and induced strong, lasting antibody responses in mice. Two doses provided robust protection against the VACV, producing higher neutralizing antibody levels than monomeric vaccines​​.	(125)
“Two-in-one” immunogen called DAM using a structure-guided strategy, which fuses the MPXV extracellular antigen A35 with the intracellular antigen M1 into a single-chain dimer.	Female BALB/c mice.	The vaccination scheme involved administering three intramuscular doses of 10 µg of DAM at 3-week intervals​.	Three weeks after the final immunization, the animals were challenged with 7 LD50 of VACV-WR. The challenge was administered intranasally following a vaccination schedule of three doses given at three-week intervals.	DAM retained the natural epitope structure, produced more robust antibody responses than co-immunization, and generated 28 times more MPXV-neutralizing antibodies than the live VACV vaccine. Aluminum-adjuvanted DAM fully protected mice from lethal VACV, and successful pilot-scale production ensured high yield and purity.	(126)

IN, intranasal; IP, intraperitoneal; SC, subcutaneous; VV-WR, Western Reserve strain of vaccinia virus.

The Subunit Smallpox Vaccine with VACV Proteins A33, B5, L1, A27, and CpG/Alum was tested in cynomolgus monkeys. In this study, Group 1 received three doses of 100 µg each at weeks 0, 4, and 12, while Group 2 received two doses at weeks 0 and 4. The challenge involved administering a lethal dose of MPXV five weeks after the final dose for the three-dose group and four weeks after the second dose for the two-dose group. The results showed that all animals in the three-dose group survived, while one animal in the two-dose group did not. The disease signs were least frequent in the three-dose group, and the vaccine was effective in enhancing neutralization titers and significantly reducing viral loads compared to controls (54).

Another study evaluated a Recombinant Subunit Smallpox Vaccine with A27V, A33V, B5V, and Adjuvants in BALB/c mice. The mice were vaccinated with 2 µg or 10 µg doses in histidine buffer and Alhydrogel, with an optional CpG adjuvant, and boosted two weeks later. Following vaccination, the mice were challenged intranasally with 1.7×10^5^ PFU of VACV. The results indicated that all groups showed minimal weight loss and high survival rates (80-100%). However, the A27V formulation, despite eliciting strong antibody responses, did not effectively protect against infection (55).

The Polyvalent Vaccine with MPXV Proteins A29L, M1R, A35R, and B6R was also tested in BALB/c mice. In this study, mice were immunized with a recombinant protein vaccine combined with 15 mg of QS-21 adjuvant, following a three-dose regimen with an initial dose on day 0, followed by boosters on days 21 and 42. The challenge involved administering a lethal dose of the MPXV strain WIBP-MPXV-001 (2.24x10^8^ PFU/mL) intranasally and intraperitoneally after the final immunization. The results showed that the vaccine significantly increased antibody levels, enhanced IFN-γ production, and improved Th1-mediated cellular immunity. Additionally, it effectively reduced MPXV replication and minimized organ damage in the mice (56).

The *Escherichia coli*-Expressed VACV A27L, B5R, and D8L vaccine was tested in BALB/c mice. In this study, mice were immunized with Escherichia coli-derived proteins using monophosphoryl lipid A and trehalose dicorynomycolate or TiterMax Gold adjuvants. The challenge involved intranasally administering 20 times the 50% lethal dose (LD50) of VV-WR (approximately 2.4x10^6^ PFU). The findings revealed that three immunizations with A27L, D8L, and B5R, or just A27L and B5R, produced strong antibodies and provided full protection against a lethal dose of VACV. The recombinant proteins effectively neutralized the virus and offered protection against lethal challenges (57).

Although recombinant protein-based vaccines against mpox represent a promising advancement in vaccine technology, there are limitations. Recombinant protein vaccines may require adjuvants to enhance their immunogenicity and often need multiple doses to achieve long-lasting immunity. Additionally, the production of these vaccines can be complex and costly. Future research should optimize these vaccines to enhance their efficacy, reduce costs, and streamline production processes, ensuring they can be widely accessible and effective in preventing mpox.

### 3.4 DNA-based vaccines

DNA vaccines work by introducing plasmids containing genes encoding specific antigens of a pathogen under the control of a eukaryotic promoter. These plasmids are administered into tissues, where the cells transcribe and translate the plasmid genes into proteins. The immune system recognizes these proteins as foreign, triggering an immune response that includes cytotoxic T-cell activation and antibody production. A key benefit of DNA vaccines is their ability to induce both cellular and humoral immune responses, which is crucial for pathogens requiring T-cell-mediated immunity, such as HIV and other intracellular agents. Genetic engineering allows the inclusion of genetic adjuvants, like immunomodulatory CpG motifs, to enhance the vaccine’s immunogenicity. DNA vaccines’ rapid design and production make them particularly useful for responding to emerging diseases and pandemics (58–60).

DNA vaccines have several advantages over other types, such as simplicity and cost-effectiveness, over traditional vaccines and viral vectors. Their design is straightforward, and they possess high stability, making them ideal for regions with limited access to refrigeration (61, 62). They are well-tolerated and can be administered repeatedly for prolonged protection without significant side effects. DNA vaccines have shown efficacy in preclinical models against various diseases, including viruses, bacteria, and parasites (63, 64). However, they typically induce less potent immune responses than conventional vaccines, so methods to improve their delivery and effectiveness are being explored, as reviewed in (65). For instance, adding immunostimulatory sequences, such as CpG and dsRNA motifs, to the transcribed region of a DNA vaccine can significantly enhance the Th1 response. These motifs can influence the production of Th1 and Th17 cytokines, improving the overall immunogenicity of the vaccine. In animal models, these modifications increase protection against pathogens like *Mycobacterium tuberculosis* (66).

A common concern with DNA vaccines is the potential integration of the introduced DNA into the host genome, which could lead to unintended genetic consequences such as insertional mutagenesis, disrupting normal cellular functions, or triggering oncogenesis (67). Nevertheless, studies on plasmid DNA vaccines in mouse models showed a shallow integration frequency into the host genome, often below detectable levels (68, 69). These studies indicated that while the risk exists, it is relatively low.

Significant advances have been made in DNA vaccines for monkeypox, particularly in designing multivalent vaccines and using intradermal electroporation techniques to improve delivery and efficacy. Some preclinical studies of DNA vaccines against monkeypox are shown in **Table 7**.

**Table 7:** Preclinical DNA vaccine candidates for mpox.

Vaccine description	Biological Model	Vaccination scheme	Challenge	Main results	Reference
Plasmid DNA encoding the monkeypox versions of the VACV proteins L1R, A27L, A33R, and B5R.	Rhesus macaques	Animals were first immunized with DNA plasmids coding for monkeypox versions of VACV proteins (L1R, A27L, A33R, B5R), delivered via ID (1 mg) and IM (3 mg). This was followed by a protein boost of 100 µg each, either in alum or with 2 mg of CpG-B ODN 2006, also given via IM.	The MPXV challenge was given via IV at a dose of 5x10^7^ PFU five weeks after the last protein boost. Combining DNA and protein, this vaccination approach led to varying levels of protection and antibody responses against monkeypox.	Group 4, which received only DNA, developed lesions and low antibody titers and did not survive. Animals given only proteins had moderate to severe disease but survived. Those vaccinated with DNA and proteins experienced mild, quickly resolving disease, high antibody levels, and controlled viremia.	(70)
This multivalent DNA vaccine contains eight VW-WR genes: A4L, A27L, A33R, A56R, B5R, F9L, H3L, and L1R.	Cynomolgous monkeys	One group of six macaques received the vaccine via ID, with each animal getting two injections of 500 µg each. Another group of four macaques was vaccinated via IM, with each receiving a single injection of 500 µg. The vaccinations occurred on three separate occasions: initially on day 0, and subsequently on days 28 and 56.	The challenge involved administering a lethal dose of the Zaire 79 strain of MPXV. The animals were infused with 2x10^7^ PFU of the virus via IV into the saphenous vein.	All vaccinated animals survived the challenge with controlled viremia and developed neutralizing antibodies, especially those vaccinated intradermally. In contrast, three out of four control animals were euthanized due to severe disease, and the remaining one showed disease signs by the end (p=0.01).	(71)
Smallpox DNA vaccine	Rhesus macaques	The vaccination schedule involved multiple sessions. The animals received the gene gun vaccinations at three specific time points: the initial and two additional booster vaccinations. These sessions occurred over a span of weeks, specifically at weeks 0, 3, and 6.	The challenge involved administering MPXV strain Zaire-79 via IV. The chosen dose for the vaccine evaluation experiment was 2x10^7^ PFU.	Monkeys vaccinated with DNA vaccines survived but continued to shed the virus. Those given the 4pox DNA vaccine showed minimal signs of mpox. Except for the negative control group, all developed neutralizing antibodies, while controls quickly succumbed to deadly hemorrhagic mpox.	(72)
Smallpox DNA vaccine	Mice BALB/c	Mice were vaccinated with a smallpox DNA vaccine using a skin electroporation device. Each received four 30 µg plasmid doses on their thighs, delivered through a microneedle array with electrical pulses. The vaccine was administered thrice at the start and weeks 3 and 5.	Intranasal VACV strain IHD-J (2×10^6^ PFU). The challenge was conducted after the final vaccination, and the mice were monitored daily for three weeks.	Vaccinated mice produced antibodies against four target immunogens and survived a lethal challenge without severe disease, whereas all control mice died within 6 to 8 days.	(73)
DNA-based 4pox vaccine targeting the L1, A27, B5, and A33 proteins.	Rabbit	The vaccination scheme described in the paper involves administering the 4pox DNA vaccine to rabbits by IM electroporation. The vaccine was administered in two different dosages. One group of rabbits received a low dose of 0.4 mg per vaccination, while another group received a high dose of 4.0 mg. The vaccine was given three times at one-month intervals.	The challenge involved a target dose of approximately 1,000 PFU of Rabbitpox Virus, administered via aerosol 28 days after vaccination.	Unvaccinated rabbits developed the severe disease and were euthanized by day 7, while all rabbits given low or high doses of the product survived and developed antibodies and controlled viremia.	(74)

IM, intramuscular; ID, intradermal; IV, intravenous; VW-WR, Vaccinia virus Western Reserve.

The comparison of DNA-based vaccine candidates across different studies highlights varied approaches, biological models, vaccination schemes, and outcomes. In one study, rhesus macaques were immunized with plasmid DNA encoding monkeypox versions of VACV proteins (L1R, A27L, A33R, B5R), delivered via intradermal (ID) and intramuscular (IM) routes, followed by a protein boost with either alum or CpG-B ODN 2006. The MPXV challenge was administered intravenously (IV) at 5x10^7^ PFU five weeks after the last protein boost. Results showed that animals receiving DNA and protein exhibited mild, quickly resolving disease, with high antibody levels and controlled viremia. In contrast, animals that received only DNA developed lesions, had low antibody titers, and did not survive, while those given only proteins experienced moderate to severe disease but survived (70). In another study, a multivalent DNA vaccine containing eight VW-WR genes (A4L, A27L, A33R, A56R, B5R, F9L, H3L, and L1R) was tested in cynomolgus monkeys. One group received ID injections, while another received IM injections, with vaccinations on days 0, 28, and 56. A lethal dose of the Zaire 79 strain of MPXV was administered intravenously. All vaccinated animals survived, exhibiting controlled viremia and developing neutralizing antibodies, particularly in the ID group. In contrast, most control animals succumbed to severe disease, with only one surviving but showing disease signs (71). A separate study involving a smallpox DNA vaccine in rhesus macaques employed multiple gene gun vaccinations at weeks 0, 3, and 6, followed by an MPXV Zaire-79 strain challenge administered intravenously. The vaccinated monkeys survived but continued to shed the virus, with the 4pox DNA vaccine group showing minimal signs of mpox and developing neutralizing antibodies. Control animals quickly succumbed to hemorrhagic mpox (72). In mice, a smallpox DNA vaccine was administered using a skin electroporation device with four 30 µg plasmid doses delivered via a microneedle array at weeks 0, 3, and 5. The challenge involved intranasal administration of the VACV strain IHD-J at 2×10^6^ PFU. Vaccinated mice produced antibodies against four target immunogens and survived the lethal challenge without severe disease, while all control mice died within 6 to 8 days (73). Finally, a DNA-based 4pox vaccine targeting the L1, A27, B5, and A33 proteins was tested in rabbits. Rabbits were vaccinated by intramuscular electroporation with either a low dose (0.4 mg) or a high dose (4.0 mg) of the vaccine, administered thrice at one-month intervals. The challenge involved aerosolized Rabbitpox Virus at approximately 1,000 PFU. Unvaccinated rabbits developed severe disease and were euthanized by day 7, while all vaccinated rabbits, regardless of dose, survived, developed antibodies, and controlled viremia (74). These studies collectively demonstrate the potential efficacy of DNA-based vaccines in eliciting strong immune responses and providing protection against MPXV and related viruses across various animal models.

Additionally, the design of multi-epitope vaccines using reverse vaccinology and immunoinformatics has led to promising vaccine candidates by in-silico simulations. These studies suggest that the candidates can generate robust immune responses and provide effective global population coverage, pending further *in vitro* and *in vivo* studies (50). The most promising candidates are designed to induce strong immune responses by presenting viral antigens to Toll-like receptors (TLRs) and the major histocompatibility complex (MHC), both crucial for effective immune system activation (75, 76).

Current approaches in developing DNA vaccines for mpox also evaluate their immunogenicity through immunological simulations to predict human body responses. These approaches aim to improve vaccine efficacy while reducing development time and associated costs (76).

### 3.5 mRNA vaccines

The new generation of messenger RNA (mRNA) vaccines offers significant advantages over previous technologies. Unlike DNA vaccines, mRNA vaccines provide superior safety since mRNA, as a temporary information carrier, does not interact with the genome, reviewed in (77). Additionally, their production process does not require handling the pathogen; instead, it uses gene sequences obtained through *in vitro* transcription from a complementary DNA (cDNA) template using a bacteriophage RNA polymerase (78). Moreover, mRNA vaccines can induce cellular and humoral immune responses (79, 80). These vaccines express proteins *in situ*, triggering balanced cellular and humoral responses (79, 80).

**Figure 4:**
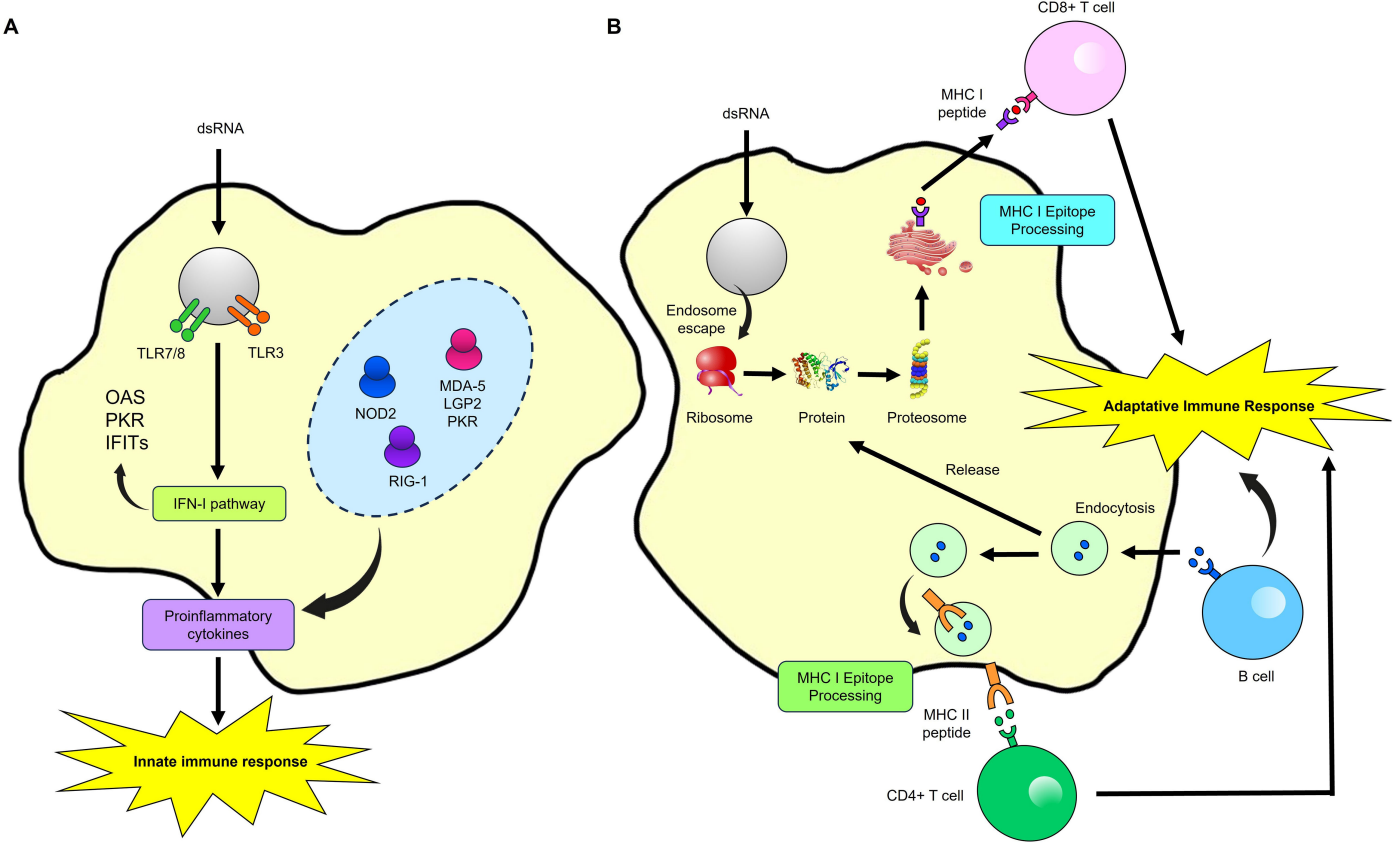
Immune Response activation by a mRNA Vaccine. **(A)** Innate immune response. When antigen-presenting cells endocytose exogenous mRNA, it is detected by TLR7/8 and TLR3 in endosomes and by LPG2, NOD2, RIG-1, NOD2, MDA-5, and PKR in the cytosol. This detection induces strong type I interferon (IFN-I) responses and the production of proinflammatory cytokines, thereby activating innate immunity. **(B)** Adaptive immune response. The protein encoded by the mRNA is released from the cell to activate B-cells. Concurrently, mRNA-encoded or re-endocytosed proteins are processed into peptides by the proteasome and presented on MHC-I molecules, activating CD4+ and CD8+ T-cells. Modified from (107).

The RNA’s ability to induce controlled cellular and humoral immune responses begins when an antigen-presenting cell endocytoses the mRNA. Double-stranded RNA (dsRNA) acts as PAMP recognized by various innate immune system receptors, such as TLRs. TLR3 and TLR7/8 in endosomes recognize the RNA and trigger a type I interferon (IFN-I) response, which is crucial for an effective antiviral response. This pathway involves proteins like RIG-I, LGP2, NOD2, RIG-1, MDA5, and PKR (81). MDA5 and PKR play critical roles in inducing IFN-I. MDA5 recognizes intracellular dsRNA, activating IFN-β transcription depending on the cell type. At the same time, PKR can induce apoptosis and is essential for MDA5-mediated signal transduction in certain viral infections, such as the VACV virus, and for regulating the immune response (82). The protein produced by the mRNA is then released from the cell to activate B-cells. Simultaneously, proteins generated from the mRNA, newly synthesized and re-endocytosed, are processed into peptides by the proteasome. These peptides are presented on MHC-I or MHC-II molecules, activating CD4+ and CD8+ T-cells, which in turn drive the activation of adaptive immunity (83–85) (**Figure 4**).

RNA vaccines provoke more inflammation because, during mRNA translation, some mRNA molecules can fold back on themselves, forming double-stranded RNA (dsRNA), a byproduct recognized by the immune system as a PAMP. This dsRNA and other PAMPs activate strong immune responses by engaging innate immune receptors. The mRNA in these vaccines is designed to instruct cells to produce specific viral proteins, which are then recognized by the immune system, triggering an adaptive immune response. Unlike DNA vaccines, which generate a more balanced Th1/Th2 immune response, RNA vaccines typically induce a Th1-dominant response. While a Th1 response effectively protects against many pathogens, it may not be optimal for all vaccine applications. The type of immune response elicited is crucial for effective protection, but the strong inflammatory response can also lead to increased toxicity, necessitating careful vaccine formulation to minimize side effects (86, 87).

Another disadvantage of RNA vaccines is their lower stability than DNA, which can reduce vaccine efficacy due to rapid degradation in the body. Effective delivery of RNA to target cells is also more challenging. DNA vaccines can integrate into the host genome and produce prolonged antigen expression. In contrast, RNA must be translated directly into the cytoplasm, a process that can be less efficient without proper formulation and delivery optimization (59). Various alternatives are being explored to improve RNA vaccine stability, such as modifying the 5’ and 3’ UTR regions, encapsulating mRNA into liposomes and other carriers, and improving production processes (79, 88–92).

Following the success of mRNA vaccines like Moderna’s mRNA-1273 and Pfizer-BioNTech’s BNT162b2 during the SARS-CoV-2 pandemic (42, 93), there is now significant interest in developing mRNA vaccines for different pathogens, including the variola virus (that causes smallpox) and MPXV to induce long-lasting humoral and cellular protection against severe diseases. These vaccines are designed to induce a Th1 immune response, correlated with protection against clinically severe diseases caused by both viruses. This approach shows great potential for developing vaccines that offer cross-protection between variola virus, MPXV, and possibly other orthopoxviruses (94).

Currently, the trend in developing mRNA vaccines against mpox focuses on creating polyvalent vaccines. Tian et al. have demonstrated that the A29L protein, a specific viral protein encoded by the orthopoxvirus genome, plays a crucial role in the immune response. A29L is highly conserved across orthopoxviruses, including MPXV and VACV, and is located on the virus’s intracellular mature virion (IMV) form. While the expression of A29L alone is sufficient to provide some protection against MPXV and VACV, Tian et al. found that the best protective results are achieved by combining multiple antigens from both the intracellular mature virion (IMV) and extracellular enveloped virion (EEV) forms of the virus (95–97). The higher level of protection provided by combining immunogens from both viral forms is likely due to antibody production at different stages of infection and through various mechanisms. However, these mechanisms are not yet fully understood.

A standout study by Sang et al. developed two quadrivalent mRNA vaccines, mRNA-A-LNP and mRNA-B-LNP, which differ from the previously mentioned vaccines by using lipid nanoparticles (LNPs) for delivery. These vaccines encode two antigens from intracellular mature virions (IMV) and two from extracellular enveloped virions (EEV) of the MPXV. In mouse models, these vaccines induced strong IgG-specific and neutralizing antibody responses against VACV, and durable cellular immunity mediated by T-cells and memory B-cells. Passive serum transfers from immunized mice successfully protected against the VACV challenge, indicating promising efficacy against mpox and other orthopoxvirus outbreaks. This cross-protection is attributed to the high conservation of surface proteins between MPXV and VACV. Compared to the current Modified Vaccinia Ankara (MVA)-based vaccine, these mRNA vaccines generate superior neutralizing and inhibitory cellular activities, along with a Th1-biased humoral immunity against MPXV antigens and their VACV counterparts (98, 99).

**Table 8** lists several preclinical studies on mRNA vaccines against mpox, highlighting the potential of mRNA vaccines to provide a flexible and rapidly adaptable platform for responding to emerging monkeypox outbreaks and other related viral challenges. **Table 8** studies used different vaccination schemes and antigen compositions, revealing the potential of mRNA vaccines as effective alternatives to traditional vaccines. For instance, mRNA-Lipid Nanoparticle Vaccines (mRNA-A-LNP and mRNA-B-LNP) administered to BALB/c mice showed that low and high doses induced robust antibody responses and nearly complete protection against lethal VACV challenge (99). Similarly, mRNA vaccines encoding M1R and A35R induced strong immune responses, with VGPox 1 and 2 outperforming VGPox 3 in virus neutralization and all vaccinated mice surviving the challenge (100). Quadrivalent MPXV mRNA vaccines also showed strong immunogenicity, producing MPXV-specific IgG, potent VACV-neutralizing antibodies, and long-lasting T-cell and memory B-cell immunity. Sera from immunized mice protected against VACV in nude mice, indicating broad, long-term protection (98). Mpox multi-antigen mRNA vaccines (mix4 and mix6) elicited strong cross-neutralizing responses, particularly with Rmix6, which showed stronger cellular immunity (96). The MPXVac-097 multivalent mRNA vaccine generated broad protective efficacy, matching the efficacy of Mix-5, and was simpler to produce (101). Finally, the quadrivalent ALAB-LNP vaccine effectively induced cellular and humoral immunity, with sera neutralizing MPXV *in vitro*, making it a promising candidate for protecting against MPXV and other orthopoxviruses (102).

**Table 8:** Preclinical mRNA Vaccine Candidates for Mpox.

Vaccine description	Model	Vaccination scheme	Challenge	Main results	Reference
mRNA-lipid nanoparticle	Mice BALB/c	The vaccination scheme involved giving BALB/c mice two IM doses of mRNA-A-LNP and mRNA-B-LNP vaccines: 40 μg on day 0 and a booster of 40 μg on day 14.	The vaccinated mice were given a lethal dose of VACV.	All MVA-immunized mice survived lethal challenges but had temporary weight loss. In contrast, mice immunized with 8 μg mRNA showed complete survival without weight loss. Mice immunized with 2 μg MPXV-mRNA also survived with minimal weight loss. Both low and high mRNA doses produced strong antibody responses, providing nearly complete protection against illness.	(99)
mRNA vaccines encoding M1R and A35R	Mice BALB/c	Mice received two IM doses of 10 μg mRNA-LNP on day 0 and day 14. On day 36, they were IN challenged with 1x10^6^ PFU VACV-WR. Researchers monitored body weight and symptoms daily, sacrificing mice if weight loss.	The challenge for vaccinated animals involved a lethal dose of VACV. exceeded 15% or at 9 days post-challenge, to evaluate vaccine protection by measuring weight changes and lung viral load.	All vaccines induced anti-A35R antibodies and T-cell responses. Only VGPox 1 and 2 produced early anti-M1R antibodies; VGPox 3 did so later. Mice with VGPox 1 and 2 had better virus neutralization than those with VGPox 3. All vaccinated mice survived a lethal virus challenge and cleared the virus from their lungs. These mRNA vaccines encoding the A35R and M1R fusion protein are highly effective and could be a safe alternative to current whole-virus vaccines.	(100)
VGPox 1 and 2 code for a fusion protein of A35R and M1R, while VGPox 3 contains separate mRNA-LNP complexes for A35R and M1R.	Mice BALB/c	The mice received two IM doses of 10 μg mRNA-LNP on days 0 and 14.	On day 36, they were challenged intranasally with 1×10^6^ PFU of VACV-WR.	All three vaccines induced early anti-A35R antibodies, but only VGPox 1 and 2 generated early anti-M1R antibodies. VGPox 1 and 2 provided better virus neutralization and antibody response compared to VGPox 3. The vaccines offered complete protection and demonstrated long-term immunity. These mRNA vaccines encoding fusion proteins of A35R and M1R are highly effective and could be a viable alternative to current whole-virus vaccines for MPXV.	(100)
Quadrivalent MPXV mRNA vaccines include mRNA-A-LNP and mRNA-B-LNP.	Mice BALB/c	Mice were immunized with two IM doses of 40 μg of mRNA-A-LNP or mRNA-B-LNP on days 0 and 14.	On day 30, mice were challenged subcutaneously with 4×10^5^ TCID_50_ of the VACV. The viral load in the mice was measured 24 h post-challenge.	Mice developed MPXV-specific IgG, potent VACV-neutralizing antibodies, and long-lasting MPXV-specific killer T-cell and memory B-cell immunity. Sera from mRNA-A-LNP and mRNA-B-LNP-immunized mice protected nude mice against the VACV challenge.	(98)
Mpox multi-antigen mRNA vaccines are called mix4 (M1, A29, B6, and A35) and mix6 (M1, A29, H3, E8, B6, and A35).	Mice BALB/c or C57BL/6 mice	Two IM doses of 1 µg or 5 µg of the multi-antigen mRNA vaccines, Lmix6, Lmix4, Rmix6, or Rmix4, were given to groups of BALB/c mice (n = 6). The first dose was given on day 0, followed by a booster dose on day 14.	The challenge for the vaccinated animals occurred 14 days after the booster dose. Mice were IP injected with a lethal dose of 5×10^6^ PFU of VACV and monitored daily for body weight and survival for 14 days post-infection. This challenge aimed to assess the protective efficacy of the vaccines against a fatal VACV infection.	Mpox multi-antigen mRNA vaccines produced strong cross-neutralizing responses against VACV. Rmix6 elicited stronger cellular immune responses than Rmix4. The M1 antigen efficiently induced neutralizing antibodies, with the top 20 antibodies targeting the same epitope as 7D11, suggesting a potential vulnerability to viral immune evasion.	(96)
Multivalent mRNA vaccine, MPXVac-097.	Mice	Animals were administered 5 µg of MPXVac-097 or Mix-5 LNP mRNA via IM to mice on days 0, 7, and 14.	Mice were given an IN dose of VACV on day 28.	MPXVac-097 triggers strong antibody and T-cell responses and protects mice from the vaccinia virus. The study compared monovalent and multivalent Mpox mRNA vaccines, including MPXVac-097, Mix-5, and single antigen LNP mRNAs. MPXVac-097 produced broad neutralizing antibodies and MPXV-specific T-cell responses, matching Mix-5’s efficacy. Its simpler production makes antigen tandem co-expression appealing. MPXVac-097 caused no significant pathology in mice.	(101)
Multi-valent mRNA vaccines.	Mice BALB/c	The vaccination scheme involved two IM doses of 7.5 μg of each mRNA vaccine (MPV-E2, MPV-M2, MPV-M4, MPV-EM6) or a placebo (Vac-Ctrl) given to BALB/c mice on days 0 and 14. Blood samples were collected on days 0, 7, 14, 28, 43, and 57 to measure antibody and T-cell responses.	The challenge for the vaccinated animals occurred on day 65 post-initial vaccination, where mice were intranasally challenged with a lethal dose of 1×10^6^ PFU of the VACV Tian Tan strain.	The vaccine generated a strong immune response, with more immunogens boosting total IgG and neutralizing activity. It also protected vaccinated mice from lethal VACV challenge.	(107)
ALAB-LNP vaccine encodes four vaccinia viral antigens (A27, L1, A33, and B5) in one molecule.	Mice BALB/c	The vaccination scheme involved administering two IM doses of the quadrivalent mRNA vaccine, ALAB-LNP, to mice. The first dose of 20 μg was given on day 0, followed by a booster dose on day 14. Blood samples were collected on days 7, 14, 28, and 42 to measure the immune response, including antibody titers and T cell activity.	On day 56, the vaccinated mice were exposed to a lethal dose of the MPXV.	This quadrivalent vaccine induced strong rodent immune responses, producing robust cellular and humoral immunity with long-lasting antibodies. Vaccinated mice’s sera also cross-reacted with antigens from multiple orthopoxviruses and neutralized the MPXV *in vitro*, suggesting ALAB-LNP as a promising candidate for protection against monkeypox and other orthopoxviruses.	(102)
MPXV-1103 vaccine encodes for four viral antigens (B6R, A35R, A29L, and M1R).	Mice BALB/c	Two doses of the MPXV-1103 mRNA vaccine were given to female BALB/c mice two weeks apart via intramuscular injection. Mice received 1, 5, or 20 μg, and immune responses were measured 10 days after the second dose. The vaccine included B6, A35, A29, and M1 proteins.	The mice were challenged by being exposed to a lethal dose of VACV eight weeks after vaccination to test their ability to survive and eliminate the virus​.	MPXV-1103 generates a stronger humoral and MPXV-specific T-cell response compared to monovalent mpox mRNA vaccines, protecting mice from a lethal vaccinia virus challenge with no live virus detected in the nasal cavities or lungs at doses as low as 1 µg. Moreover, complete blood counts and tissue analysis in mice given 5 µg and 20 µg doses showed no observable pathological changes after two doses.	(127)
Two bivalent MPXV mRNA vaccines, designated LBA (B6R-A29L) and LAM (A35R-M1R), and a quadrivalent mRNA vaccine, LBAAM (B6R-A35R-A29L-M1R)	Mice BALB/c	The mice received two intramuscular doses of the mRNA vaccine, spaced two weeks apart, each containing 20 µg of the vaccine.	The mice were immunized with two doses of 20 µg of the mRNA vaccine, given intramuscularly at a 2-week interval. Thirty days after the initial vaccination, the mice were challenged with 1x10^6^ PFU of vaccinia virus via intranasal administration.	After two doses, the mRNA vaccines induced strong antibody and cellular responses, providing complete protection against lethal VACV infection in mice. Mice given the LBA&LAM or LBAAM vaccines had minor weight loss but recovered quickly, with significantly less lung tissue damage than the LNP control groups.	(128)
Two multivalent mRNA vaccine candidates: a quadrivalent vaccine (BNT166a), which encodes the MPXV antigens A35, B6, M1, and H3, and a trivalent vaccine (BNT166c), which includes A35, B6, and M1 but excludes H3.	Macaques	The BNT166 vaccine was administered in two intramuscular doses: the initial dose on day 0 and a booster on day 21.	The animals were challenged with VACV after receiving two doses of the BNT166 vaccine on days 0 and 21. Three weeks after the second dose, mice were exposed to a lethal dose of 5 × 10^4^ PFUs of VACV​​.	Both vaccine candidates induced strong T cell and IgG responses in mice, including neutralizing antibodies against MPXV and vaccinia virus. In challenge studies, BNT166a and BNT166c provided full protection, with BNT166a also fully preventing death and lesions in a lethal clade I MPXV challenge in macaques.	(129)

IM, Intramuscular; IN, Intranasal; IP, Intraperitoneal; VACV-WR, Western Reserve strain of the Vaccinia virus; LNP, Lipid nanoparticles.

The findings across these studies highlight the potential of mRNA vaccines in providing robust protection against MPXV and related viruses. Including multiple antigens and using lipid nanoparticle formulations were critical in enhancing the vaccines’ protective efficacy and long-term immunity. These mRNA-based approaches offer a viable and potentially safer alternative to traditional whole-virus vaccines, paving the way for further development and clinical evaluation of mRNA vaccines against monkeypox and other orthopoxvirus infections.

## 4 Future strategies for enhancing mpox vaccine efficacy and stability

Several advanced strategies are being explored to address the limitations of current mpox vaccines. Incorporating immunostimulatory sequences such as CpG and dsRNA motifs into DNA vaccines has significantly enhanced their immunogenicity by promoting stronger and more targeted immune responses (66). Improved delivery systems, such as electroporation and other cutting-edge technologies, are also being used to increase the efficacy of DNA vaccines by ensuring more efficient delivery and uptake of the vaccine into the host cells (76).

Stability is a critical challenge for mRNA vaccines. Modifying the 5’ and 3’ UTR regions and encapsulating mRNA into liposomes are promising approaches to enhance their stability and efficacy. These modifications help protect the mRNA from degradation and improve its delivery to target cells, enhancing the vaccine’s effectiveness (79).

An underexplored alternative for MPXV vaccine design is the synergistic effect of generating humoral and cellular responses by combining nucleic acid vaccines with the same recombinant antigens. This combination could enhance immune responses and offer flexibility for rapid adaptation to new virus variants (103).

Developing polyvalent vaccines expressing multiple viral antigens can also provide broader and more robust protection against orthopoxvirus strains. These polyvalent vaccines can elicit an immune response capable of neutralizing diverse strains of the virus, which is particularly crucial for combating evolving and emerging pathogens (95, 96, 102). Combined with advances in immunoinformatics, these approaches promise to revolutionize vaccine design (104).

Overall, developing polyvalent vaccines, immunoinformatics for epitope optimization, and combining nucleic acid vaccine technologies represent promising strategic approaches to improve protection against monkeypox and other orthopoxviruses. Future research and clinical trials will be essential to validate these approaches and ensure their efficacy and safety in human populations. Integrating these scientific advancements could herald a new era in viral disease prevention, with more effective vaccines adaptable to global public health needs.

## 5 Conclusions

The recent global mpox epidemic and the emergence of more virulent strains in the Democratic Republic of the Congo highlight the urgent need for new, effective, and safe vaccines against orthopoxviruses. Although effective in eradicating smallpox, first-generation vaccines based on live, replicating VACV and second-generation vaccines, which were slightly modified but still based on live VACV, present serious complications and limitations for widespread use due to significant adverse reactions and production challenges. Third-generation vaccines, such as MVA-BN, use a highly attenuated, non-replicating VACV strain and are associated with fewer side effects. However, these vaccines show lower immunogenicity in previously unvaccinated populations, raising concerns about their efficacy in future outbreaks. Additionally, discontinuing smallpox vaccination programs has reduced global immunity against orthopoxviruses, increasing the risk of severe infections and zoonotic transmission (32, 102, 105).

This underscores the importance of continued research and development of new mpox vaccines to prevent potential future outbreaks. However, scientific, logistical, regulatory, and socioeconomic challenges must be addressed for efficient immunization. Ensuring vaccine safety and efficacy is crucial. Large-scale production and efficient distribution of vaccines are essential, especially for mRNA vaccines that require advanced infrastructure and ultra-low temperature storage, limiting their availability in resource-limited regions. The high development and production costs of mRNA vaccines may also affect their accessibility in low-income countries. Additionally, public acceptance is vital; misinformation and distrust can hinder vaccination campaigns, necessitating communication and education efforts to inform about their safety and efficacy.

This work emphasizes the urgent need to maintain momentum in researching and developing new mpox vaccine candidates, ensuring they are safe and effective. Persistence in this field is essential to tackle our scientific, logistical, regulatory, and socioeconomic challenges. We can ensure population protection against future infectious disease outbreaks and potential bioterrorist threats through a continuous commitment to improving vaccines.
